# High-risk infant follow-up: current practice and factors determining eligibility

**DOI:** 10.1038/s41390-025-04154-2

**Published:** 2025-06-10

**Authors:** Danielle Clifford, Sylke Steggerda, Nathalie Maitre, Linda S. de Vries, Deirdre M. Murray

**Affiliations:** 1 INFANT Centre, University College Cork, Cork, Ireland; 2 Department of Paediatrics and Child Health, University College Cork, Cork, Ireland; 3 Leiden University Medical Centre, Leiden, the Netherlands; 4 Emory University and Children’s Healthcare of Atlanta, Atlanta, Georgia; 5 Department of Paediatrics, Cork University Hospital, Cork, Ireland; 6 ARNAS Civico Di Cristina Benfratelli, Palermo, Italy; 7 Department of Neonatology, Paediatric Intensive Care and Neuropaediatrics, Medical University of Vienna, Vienna, Austria; 8 Dalhousie University, Division of Neonatal-Perinatal Medicine, IWK Health, Nova Scotia Provincial Perinatal Follow Up Program, Halifax, NS Canada; 9 McMaster Children’s Hospital, Hamilton, ON Canada; 10 Universidade Federal do Rio Grande do Sul and Hospital de Clínicas de Porto Alegre, Porto Alegre, Brazil; 11 Nagoya University Graduate School of Medicine in Nagoya, Nagoya, Japan; 12 Neonatal Neurology, Fetal Neonatal Neuroscience, Children’s Hospital of Philadelphia/Perelman School of Medicine at University of Pennsylvania, Philadelphia, PA USA; 13 Federal University of Rio Grande do Sul- Neonatal Section- Hospital de Clinicas de Porto Alegre, Porto Alegre, Brazil; 14 Children’s Hospital Colorado/University of Colorado Anschutz Medical Campus, Aurora, CO USA; 15 Departamento de Pediatria, Hospital de Santa Maria, ULSSM; Clínica Universitária de Pediatria, Faculdade de Medicina, Universidade de Lisboa, Lisboa, Portugal; 16 University College London Hospital (UCLH) and London School of Hygiene and Tropical Medicine (LSHTM), London, UK; 17 Division of Child Neurology, Department of Pediatrics, University of Utah, Salt Lake City, UT USA; 18 Gävle Regional Hospital, Region Gävleborg, Sweden; 19 Department of Women’s and Children’s Health, Uppsala University, Uppsala, Sweden; 20 Serviço de Neonatologia, Unidade Local de Saúde de Coimbra, Coimbra, Portugal; 21 Department of Pediatrics, Northwestern University Feinberg School of Medicine, Chicago, IL USA; 22 Department of Neonatology, Bai Jerbai Wadia Hospital for Children, Mumbai, Maharashtra India; 23 Royal College of Surgeons Ireland, Dublin, Ireland; 24 Pediatrics, Division of Child Neurology, Nationwide Children’s Hospital and The Ohio State University College of Medicine, Columbus, OH USA; 25 Pediatric Neurology, Cleveland Clinic, Cleveland, OH USA; 26 Phoenix Children’s, Phoenix, AZ USA; 27 Department of Neonatology, University and Polytechnic Hospital La Fe, Valencia, Spain; 28 Department of Neonatology, Wilhelmina Children’s Hospital, University Medical Center Utrecht, Utrecht University, Utrecht, the Netherlands; 29 Division of Neonatology, Central Teaching Hospital of Bolzano/Bozen, Bozen, Italy; 30 Cork University Maternity Hospital, Cork, INFANT Centre, University College Cork, Cork, Ireland; 31 The Hospital for Sick Children, Division of Neonatal Neurology, Toronto, ON Canada; 32 Helsinki University Hospital, Pediatrics, Finland; 33 Department of Neonatal and Pediatric Intensive Care, Division of Neonatology, Erasmus University Medical Center-Sophia Children’s Hospital, Rotterdam, the Netherlands; 34 INFANT Research Centre, University College Cork, Cork, Ireland; 35 Department of Paediatrics, University College Cork, Cork, Ireland; 36 University of Montreal, CHU Ste-Justine and Azrieli Research Center, Quebec, QC Canada; 37 University of Puerto Rico Science Medical Campus, San Juan, Puerto Rico; 38 Johns Hopkins University School of Medicine, Baltimore, MD USA; 39 Hospital das Clínicas e Departamento de Pediatria da Faculdade de Medicina da Universidade Federal de Minas Gerais, Minas Gerais, Brazil; 40 Brenner Children’s Hospital, Atrium Health Wake Forest Baptist, Wake Forest School of Medicine in Winston-Salem, Winston-Salem, NC USA

## Abstract

**Background:**

High-risk infant follow-up (HRIF) lacks universal definition. The aim of this study was to report current practice and factors used to identify eligibility for HRIF, yielding information which may provide a basis for future consensus.

**Methods:**

A survey was prepared for a workshop at the 15th International Newborn Brain Conference on prediction of outcome, which was subsequently distributed to all attendees (*n* = 426).

**Results:**

Follow-up was offered by 97% of respondents (*n* = 113/116). HRIF was offered to infants born <28 weeks by 47%, to those <32 weeks by two-thirds (66%) and to preterms based on neuroimaging by 54%. For infants born full-term, HRIF was offered by 88% in neonatal encephalopathy (NE) and 86% in neonatal stroke. HRIF continued most frequently until 24 months corrected (33.6%). For guiding prognosis in preterm infants, 22% (*n* = 25) selected neuroimaging as the most important factor. For NE, 54% (*n* = 63) selected neuroimaging findings as the most important factor in guiding prognosis and 14% (*n* = 16) selected EEG/aEEG. Social factors are not considered by 46% in determining HRIF eligibility.

**Conclusion:**

Significant variability in HRIF exists, without consensus. Awareness of factors predicting prognosis and the importance of social risk-factors must improve to allow accurate identification of those at highest risk. This information may act as a basis for future consensus on HRIF.

**Impact:**

- There is no clear consensus on eligibility or duration of high-risk infant follow-up. We report current practice in, and factors used to identify eligibility for same, amongst attendees of the International Newborn Brain Conference.
- This information on international practice may provide a basis for future consensus.
- Given the importance of accurate prognostication in risk-stratification, we report participants’ awareness of the most important factors guiding prognosis.
- A disconnect between the impact of social factors on outcome and their consideration for eligibility of high-risk infant follow-up is noted. We propose the need for guidelines on follow-up of socially disadvantaged, medically high-risk infants.

## Introduction

Factors such as prematurity and neonatal encephalopathy (NE) increase the risk of neurodevelopmental delay and disability.^1–5^ High-risk infant follow-up (HRIF) after neonatal care is generally offered to these children and their families to provide close surveillance, allowing early detection and intervention if required. However, the definition of ‘high-risk’ is not universal and eligibility varies in practice.^6–11^ Many recommendations on HRIF, including guidelines from the American Association of Pediatrics (AAP), were written for infants who were born preterm or with a low birthweight, without explicitly addressing other conditions such as NE, perinatal stroke, congenital cardiac disease or brain malformations which can also put infants at high-risk of neurodevelopmental impairment.^12,13^ European Standards of Care for Newborn Health on follow-up and continuing care additionally recognise grade 2–3 hypoxic-ischaemic encephalopathy (HIE) and severe foetal growth restriction as significant risk factors requiring ‘targeted structured follow-up’.^14^ Once assigned to HRIF, the schedule of follow-up, type of assessments during follow-up and duration of follow-up differs, with no universal standard of care and varying approaches between centres and clinicians.^6,7,10,11,15^

Prognosis and early detection of developmental delay are an important part of HRIF and help to target the type of surveillance and intervention required. In addition, HRIF programmes can assist families in accessing relevant information and supports. In the premature infant, predicting prognosis soon after birth is challenging and cannot be based on gestational age alone.^2,16–18^ Many factors have been investigated for their prognostic value including laboratory-based biomarkers, neuroimaging and standardised examinations.^19,20^ Varying combinations of these have also been explored, with a growing interest in machine learning techniques in recent years, allowing us to examine even non-linear relationships.^21–24^ Neuroimaging has been found to have the most predictive value for motor, cognitive and language development, with several tools published, and imaging findings warrant consideration when determining which infants are most ‘high-risk’ for developmental sequelae.^25–27^

With regards to term infants with hypoxic-ischaemic encephalopathy (HIE) the introduction of therapeutic hypothermia has significantly improved outcomes over the past two decades, such as death or long-term major neurodevelopmental disability.^28^ However, HIE remains an important cause of neurodisability in term infants, with adverse outcomes across the grades of severity, including death and motor or cognitive impairment.^4,28^ For HIE, EEG and aEEG findings have been shown to be an excellent early marker of neurodevelopmental impairment including cognitive and motor outcome, diagnosis of cerebral palsy, GMFCS level, and death.^29–32^ Other means of predicting outcomes such as clinical details, lab-based biomarkers or neuroimaging are of less individual prognostic benefit and previously published predictive models are not yet ready for clinical use.^20,30–34^ Collectively however, individualised neuroprognostication is possible for outcomes such as cerebral palsy, cognition, epilepsy and cortico-visual impairment using expert consensus on MRI findings in combination with clinical, laboratory and EEG parameters.^35^

Less well documented is the influence that socioeconomic status has on prognosis for these high-risk infants. American Association of Pediatrics (AAP) guidelines on discharge of the high-risk infant from hospital highlight the significance of social risk factors, and there have been calls to systematically screen for these in HRIF programmes, as in all paediatric care.^36,37^ However, AAP guidelines on monitoring preterm infants’ neurodevelopment after discharge focus on medical factors such as gestational age, necrotising enterocolitis, broncho-pulmonary dysplasia and intraventricular haemorrhage without specifically addressing the increased risk associated with social factors in their Risk Stratification Framework or algorithm.^13^ NICE guidelines highlight maternal socioeconomic status as a risk factor for cognitive and developmental outcomes of preterm infants but again do not take this into consideration when recommending a specific follow-up schedule.^38^ While the research focus and clinical improvements have centred around medical factors which affect the outcomes of these children, the social factors impacting their trajectories remain under-investigated and under-appreciated, particularly in the cohort of term infants with NE. This has been noted as one of the areas of HRIF most in need of attention.^39^ The European Standards of Care for Newborn Health, which has parent and patient involvement, recognise the risk of cognitive impairment is “highest for extremely preterm births or those with perinatal asphyxia and most severe in those with additional social disadvantage”.^40^ Using machine learning techniques, predictive algorithms have also begun to highlight the importance of socio-economic factors on high-risk infants’ outcomes, such as cognitive delay at two years corrected.^24^ The concept of ‘follow-through’ has been suggested to highlight the importance of supporting families with non-technical aspects of care beyond their stay in the NICU.^41^

Despite much research and recommendations, little is documented about current practice of HRIF around the world, which likely varies between countries, centres and even clinicians.^7^ The aim of this study was to report current practice and factors used to identify eligibility for high-risk infant follow-up amongst attendees at an international conference focused on neonatal brain care. We hypothesised that the information gained from this survey could provide clinicians with information on the differences and commonalities in international practice, which may offer a basis for future consensus.

## Methods

The 15th International Newborn Brain Conference was held in Cork, Ireland on February 28th – March 2nd, 2024. This was attended by 426 participants, 299 in-person and 127 online. Attendees were from 50 different countries worldwide, with, respectively, the United States, Ireland, the United Kingdom, Canada, Australia, the Netherlands, Italy, Belgium, Germany and Austria most frequently represented. A survey was prepared by authors DM, NM, SS, LdV as part of a workshop entitled ‘Early Prediction of Outcome in Term and Preterm Infants - Best Tools’. Following the workshop, the survey was distributed to all attendees of the conference. A copy of the survey is included in the supplementary material.

Consent was obtained as follows; participants were given three options to select from, firstly ‘I am happy to take the survey but please do not use my answers in your publication’ *n* = 2, second ‘I am happy for you to use my answers in the publication but do not wish to co-author’ *n* = 41, or finally ‘I would like to co-author and am happy to edit and contribute to the manuscript preparation’ *n* = 75. The Emory Institutional Review Board (IRB) determined that this project did not require IRB review because it was deemed not ‘research’ as defined in the federal regulations and should be designated as ‘not human subjects research’.

Descriptive statistics were used to analyse the data which was exported from Microsoft forms. IBM SPSS v28® statistical analysis software (IBM Corp. released 2012 Armonk, NY) was used to perform testing.

## Results

A total of 118 responses were received from participants giving a 28% response rate from overall conference attendees. Two did not wish for their answers to be used for publication. The remaining 116 responses are included in the current analysis.

Of the respondents included, 62 (53%) were neonatologists, 20 (17%) were trainees/junior doctors, 18 (16%) were paediatric neurologists, 4 (3%) were paediatricians and 12 identified as ‘other’ which was comprised mainly of occupational therapists, physiotherapists, clinical psychologists, nurse practitioners and physician assistants. The level of experience was well distributed, with all career stages represented, from student to retiree; years’ experience reported were 1–5 in 25%, 5–10 in 17%, 10–15 in 22%, 15–20 in 23% and 12% answered ‘too old to remember’.

**Fig. 1:**
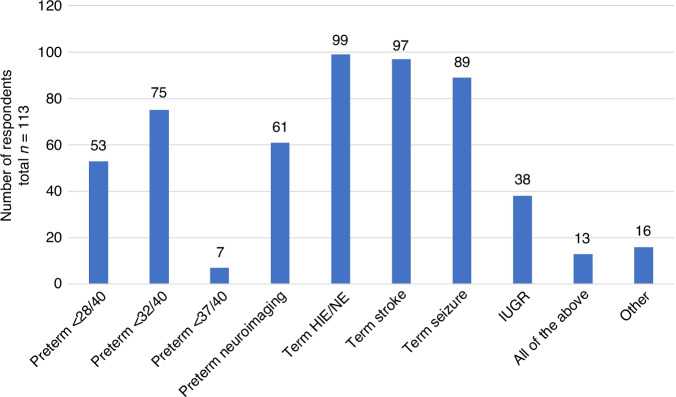
Criteria reported by respondents as being those used for determining eligibility for high risk infant follow up in their centre. Respondents were asked to tick yes to all that applied. IUGR intrauterine growth restriction, HIE hypoxic ischaemic encephalopathy, NE neonatal encephalopathy.

Neurodevelopmental follow-up was offered by 97% (*n* = 113) of participants’ centres; 14% (*n* = 16) to all infants admitted to the NICU and 84% (*n* = 97) to high-risk infants only. When asked regarding follow-up for ‘high-risk infants only’, 47% (*n* = 53) reported offering follow-up to preterm <28 weeks gestation, two-thirds (66%, *n* = 75) for preterm <32 weeks gestation and 7% (*n* = 7) for all preterm <37 weeks gestation (Fig. 1). Neuroimaging findings were considered by 54% (*n* = 61). In relation to term infants, most followed-up those with HIE (hypoxic-ischaemic encephalopathy)/NE (88%, *n* = 99), stroke (86%, *n* = 97) and seizures (79%, *n* = 89). One-third (33%, *n* = 38) offer HRIF for those with intra-uterine growth restriction. Follow-up was offered in all instances listed above by 12% (*n* = 13) and 14% (*n* = 16) report follow-up for ‘other’ reasons not specified.

Duration of follow-up was most frequently until 24 months corrected age (34%, *n* = 39), followed by 5 years (20%, *n* = 23) and 36 months (10%, *n* = 12), with a small number offering follow up to 8 years (10%, *n* = 11).

With regards to prognostication in preterm infants, the single most important factor in guiding prognosis was felt to be the clinical course of the infant by 47% (*n* = 54), neuroimaging by 22% (*n* = 25), structured neurological examination by 21% (*n* = 24), family circumstance by 10% (*n* = 12) and EEG/aEEG by 1 respondent. Less than 20% (*n* = 17) of consultants and 30% (*n* = 6) of trainees/junior doctors selected neuroimaging as the most important factor in the prognosis of preterm infants. For term infants with NE, 54% (*n* = 63) felt that neuroimaging findings were the single most important factor in guiding prognosis, 22% (*n* = 25) thought it was structured neurological examination, 14% (*n* = 16) EEG/aEEG, 4% (*n* = 5) family circumstance, 3% (*n* = 4) Sarnat score and 3% (*n* = 3) blood-based biomarkers. Only 10 consultants (12%) and 5 trainee/junior doctors (25%) selected EEG/aEEG as the most prognostic factor.

**Fig. 2:**
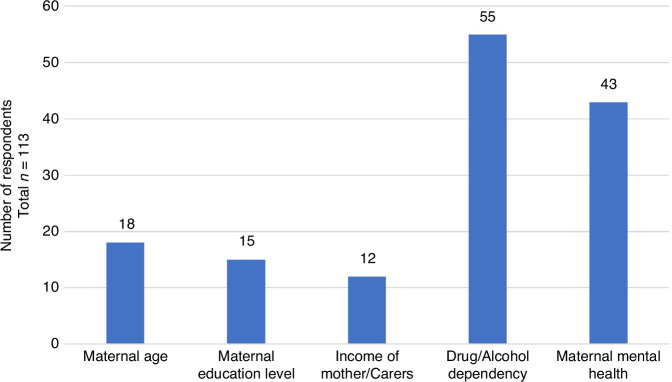
Social risk factors  reported by respondents as being used in their service to decide on eligibility for high risk infant follow up. Multiple answers were allowed.

Social risk factors were taken into account by 40 participants’ centres (35%) when deciding on follow-up and 18% (*n* = 20) report it is determined by the attending physician. 3% were unsure if social risk factors are considered when deciding on follow-up and 45% (*n* = 52) reported they are not. Risk factors taken into account included substance dependency in 49% (*n* = 55), maternal mental health in 38% (*n* = 43), maternal age in 16% (*n* = 18), maternal level of education in 13% (*n* = 15) and maternal/carer income in 11% (*n* = 12) (Fig. 2).

## Discussion

This study shows a wide variety of practices for HRIF. The majority of participants reported follow-up until at least 2 years corrected age, with 30% following to school age. Previous HRIF improvement projects have cited time, knowledge and perceived benefit as concerns regarding the establishment of HRIF.^42^ While we cannot ameliorate the universally limited resource of a clinicians’ time, it is important to highlight knowledge and perceived benefit as rectifiable barriers in granting these infants access to the required follow-up. This survey was a snapshot of current practice and did not investigate the underlying reasons for responses given. This survey builds on work carried out by the US-based High-Risk Infant Follow Up Networking Group.^7^ This qualitative study identified challenges and opportunities for the development of widespread HRIF in the US. Our survey results confirm their findings from an international cohort and support the need for consensus guidelines for HRIF.

This survey was of an experienced and motivated group of clinicians and academics from across the globe, attending a conference of subspecialist interest focused on the neonatal brain. There was a wide variation in the selected ‘most important’ factor for guiding prognosis with only 21.6% choosing the most precise marker in preterm infants, and just 13.8% selecting EEG/aEEG for term infants with NE/HIE. Neuroimaging is recommended for prognostication in preterm infants, with MRI at term equivalent age providing valuable predictive information.^43,44^ While most of those surveyed felt that neuroimaging is the best predictor of outcome following NE/HIE, and it has been shown to be of benefit, previous studies are inconsistent with numerous scoring systems in use and variability in time of scanning.^30,31,45^ MRIs performed earlier, i.e. in the first week of life, have been shown, in a meta-analysis, to be more predictive of outcome for infants with HIE than those performed later, where outcome included motor, cognitive or language development, diagnosis of cerebral palsy, GMFCS level and death.^30^ EEG/aEEG has been reported as an excellent predictor of outcome following HIE, but despite this, a very small number of respondents selected this as the most important prognostic indicator for these infants.^29–32^ In clinical practice, a combination of tools is often used to inform prognostication.^35^

There is mounting evidence that social risk factors are an important determinant of outcome, specifically cognitive outcome, not only for preterm infants but also for term infants with NE. At present many centres continue to offer ‘high-risk’ follow-up based primarily on medical risk factors, and do not offer follow-up to those at highest risk due to their social circumstances. The reasons for this are likely to be manifold; such as lack of resources, lack of training, and lack of clear guidelines. In settings where social deprivation levels are highest these factors may be even more pronounced.

Clinicians, parents of preterm infants and ex-preterm adults all perceive physical health as a more important determinant of quality of life than factors such as finances, education or intelligence in ex-preterm adults, highlighting widespread under-appreciation of socio-economic factors.^46^ Our findings would seem to echo this, with higher rates and more consistent follow-up reportedly offered to those with medical diagnoses or complications than to those with socio-economic risk factors. Among those who do offer follow-up based on social circumstances, this is primarily related to maternal substance abuse or mental health issues, with less awareness of the significance that maternal education has in predicting the child’s outcome. In preterm infants, maternal education has been shown to be as significant as brain injury in determining cognitive outcomes, particularly in the longer term.^47–49^ The effects of maternal education have been demonstrated in Low-Moderate Income Country (LMIC) settings also, predicting both language and motor development.^50^ A systematic review reported fourteen of fifteen studies showed a significant effect of socio-economic demographics on cognitive outcomes.^51^ Factors such as non-native family language, parental education level and neighbourhood deprivation have been shown to have an effect on cognitive outcomes, speech, language and communication difficulties as well as executive function skills in ex-preterm children.^17,24,52^ The significance of social disadvantage and parental education persists into childhood and through adolescence in children born extremely preterm, with a more influential effect on cognitive outcomes than gestational age.^16,53^ Furthermore, the gap between sociodemographic groups has been shown to increase with age, and this effect is even more pronounced in those with brain injury, highlighting the vulnerability of these children who are medically high-risk but with the additional complication of socio-economic status.^48^ With regards to term infants, there is an increased likelihood of adverse outcomes in countries where groups may be marginalised by race or ethnicity. For example, Black and Hispanic infants in San Diego with HIE are more likely to have a tracheostomy, gastrostomy or a diagnosis of cerebral palsy by one year of life.^54^ Conversely, it is also important to consider how better resources impact outcomes. Notably, higher parental education or richer literacy environment, measured by the number of books in the household, has been associated with improved cognitive outcomes in infants with HIE, with a more significant effect on outcomes than brain injury score.^55^ We propose the need for guidelines on follow-up of socially disadvantaged medically high-risk infants.

The strengths of this study include the wide range of experience represented and the international cohort surveyed, with attendees from more than 50 countries worldwide. The limitations include the significant bias inherent in our population selection as the conference attendees are likely to be those most motivated to learn and most interested in the newborn brain and HRIF. This may represent a higher level of awareness of factors affecting neurodevelopmental outcomes of high-risk infants than one would expect from a population of general neonatologists or general paediatricians. We also did not assess inter-site variability or the differences between developed and LMIC. Some sites may be over-represented if there was a group of participants from a single site. Attendees of the conference were predominantly from higher-income countries, and this may be reflected in the survey respondents. Participants were not asked what tools they use for follow-up or how they assess or define outcome. This was a short survey and was not designed to explore the underlying reasons for the answers given. Further qualitative study of the topic such as that carried out by the High-Risk Infant Follow Up Networking Group may be helpful in shedding further light on this area.^7^

## Conclusion

This survey highlights the significant variability in practice of HRIF and the need for a universal consensus on eligibility and duration of HRIF, with parent and patient involvement. There is also a necessity for increasing awareness of the factors predicting prognosis and the importance of social risk factors in high-risk infants, to allow optimal identification of those infants most in need of HRIF.

## Supplementary information

Supplementary Information

## Data Availability

The data generated and analysed during the current study are available from the corresponding author on reasonable request.

## Licensing Statement

This publication has emanated from research supported in part by a grant from Science Foundation Ireland (grant number 22/SPP/11074) and the Cerebral Palsy Foundation. For the purpose of Open Access, the author has applied a CC BY public copyright licence to any Author Accepted Manuscript version arising from this submission.
