# Supplementary material for: High-risk infant follow-up: current practice and factors determining eligibility
Source: Pediatr Res. 2025 Jun 10;99(1):203–8. doi: 10.1038/s41390-025-04154-2 (PMC12920091; doi:10.1038/s41390-025-04154-2)
Supplement: Supplementary file 1 — Supplementary Information [file 41390_2025_4154_MOESM1_ESM.pdf]

Survey; Early Prediction of Outcome in Term and Preterm Infants

1. Thank you for participating in our workshop and survey. We will collect responses and hope to publish the results to inform practice
    a. I would like to co-author and am happy to edit and contribute to the manuscript preparation
    b. I am happy for you to use my answers in the publication, but I don't want to co-author
    c. I am happy to take the survey but please don’t use my answers in your publication

2. If you are happy to co-author and contribute, please insert your email here

    ____

3. My current role
    a. Neonatologist
    b. Paediatrician
    c. Paediatric Neurologist
    d. Trainee/Junior Doctor
    e. Neonatal Nurse
    f. Paediatric Nurse
    g. Other ________________

4. My level of experience in neonatal/paediatric care
    a. 1-5 years
    b. 5-10 years
    c. 10-15 years
    d. 15-20 years
    e. >20 years

5. In my centre neurodevelopmental follow up is offered to the following
    a. No one
    b. High-risk infants only
    c. All infants admitted to the NICU

6. If you offer high-risk follow-up only: which of the following are included in ‘high risk’
    a. Preterm <28 weeks gestation
    b. Preterm <32 weeks gestation
    c. Preterm <37 weeks gestation
    d. Preterm based on Brain Imaging
    e. Term with HIE/NE
    f. Term with stroke
    g. Term with seizures
    h. Intrauterine Growth Restriction
    i. All of the above

j. Other _________________

7. In your centre, are social risk factors taken into account when deciding on need for follow up?
    a. Yes
    b. No
    c. Depends on the attending physician
    d. I don't know

8. If yes, what social risk factors do you take into account?
    a. Age of mother
    b. Education level of mother
    c. Income of mother/carers
    d. Drug/alcohol dependency
    e. Maternal mental health
    f. None of the above
    g. Other __________________

9. Up to what age/corrected gestational age (cGA) do you complete neonatal follow up?
    a. 3 months cGA
    b. 6 months cGA
    c. 12 months cGA
    d. 24 months cGA
    e. 36 months
    f. 5 years
    g. 8 years
    h. Other __________________

10. 10. Which of the following do you think is MOST important in guiding prognosis in preterm infants
    a. Clinical course of the infant
    b. Brain imaging
    c. EEG/aEEG findings
    d. Family circumstance
    e. Structured neurological examination

11. Which of the following are the MOST important in guiding prognosis in term NE?
    a. Sarnat score
    b. Brain imaging findings
    c. aEEG/EEG
    d. Blood-based biomarkers
    e. Family circumstance
    f. Structured neurological examination
